# Common Bacterial Infections and Risk of Dementia or Cognitive Decline: A Systematic Review

**DOI:** 10.3233/JAD-200303

**Published:** 2020-08-18

**Authors:** Rutendo Muzambi, Krishnan Bhaskaran, Carol Brayne, Jennifer A. Davidson, Liam Smeeth, Charlotte Warren-Gash

**Affiliations:** a Faculty of Epidemiology and Population Health, London School of Hygiene and Tropical Medicine, London, UK; b Cambridge Institute of Public Health, Cambridge University, Cambridge, UK

**Keywords:** Cognition, dementia, infections, prevention, systematic review, Systematic review registration number: CRD42018119294, registered in December 2018

## Abstract

**Background:**

Bacterial infections may be associated with dementia, but the temporality of any relationship remains unclear.

**Objectives:**

To summarize existing literature on the association between common bacterial infections and the risk of dementia and cognitive decline in longitudinal studies.

**Methods:**

We performed a comprehensive search of 10 databases of published and grey literature from inception to 18 March 2019 using search terms for common bacterial infections, dementia, cognitive decline, and longitudinal study designs. Two reviewers independently performed the study selection, data extraction, risk of bias and overall quality assessment. Data were summarized through a narrative synthesis as high heterogeneity precluded a meta-analysis.

**Results:**

We identified 3,488 studies. 9 met the eligibility criteria; 6 were conducted in the United States and 3 in Taiwan. 7 studies reported on dementia and 2 investigated cognitive decline. Multiple infections were assessed in two studies. All studies found sepsis (*n* = 6), pneumonia (*n* = 3), urinary tract infection (*n* = 1), and cellulitis (*n* = 1) increased dementia risk (HR 1.10; 95% CI 1.02–1.19) to (OR 2.60; 95% CI 1.84–3.66). The range of effect estimates was similar when limited to three studies with no domains at high risk of bias. However, the overall quality of evidence was rated very low. Studies on cognitive decline found no association with infection but had low power.

**Conclusion:**

Our review suggests common bacterial infections may be associated with an increased risk of subsequent dementia, after adjustment for multiple confounders, but further high-quality, large-scale longitudinal studies, across different healthcare settings, are recommended to further explore this association.

## INTRODUCTION

Dementia is a major global health challenge. Worldwide, approximately 50 million people are currently living with dementia, and this number is projected to rise to over 152 million by 2050 [1]. Given the increasing life expectancy and absence of a cure or disease-modifying therapy, dementia prevention has become a public health priority [2]. Recent evidence suggests modifiable risk factors may have contributed to a decline in the age-specific incidence of dementia in Europe and the United States [3–9], highlighting the importance of identifying and targeting modifiable risk factors, as age increases. Bacterial infections have been identified as one potentially important risk factor for dementia[10, 11].

Symptomatic bacterial infections such as pneumonia and urinary tract infections are common, and complications frequently occur among older people. One of the hallmark complications of common bacterial infections is delirium; a serious neuropsychiatric syndrome characterized by acute cognitive dysfunction and inattention [12]. Delirium is strongly associated with an increased risk of subsequent cognitive decline and dementia [13–15]. Increasing evidence suggests cognitive impairment may persist for years after sepsis hospitalization [16, 17]. However, it is unclear whether there are long term effects of common infections on cognition and dementia, independent of delirium.

Previous reviews have investigated the role of bacterial pathogens on Alzheimer’s disease; however, evidence is inconsistent, and the exact nature of this association remains unclear. In a meta-analysis of predominantly case-control studies Maheshwari and Eslick found that *Chlamydia pneumonia*, a bacterium responsible for pneumonia and other respiratory tract infections, was associated with a five-fold (OR 5.66; 95% CI 1.83–17.51) increased occurrence of Alzheimer’s disease [11]. However, due to the cross-sectional nature of the data collected in these studies, it was not possible to assess temporality. Additional drawbacks of this meta-analysis included differences in methodology and the relatively small sample sizes of the studies included (total sample size ranging from 2 to 200 samples). Furthermore, other bacterial microorganisms have also been implicated in previous and subsequent reviews [10, 18] and differing conclusions have been drawn on the role of *Chlamydia pneumonia* with Alzheimer’s disease as evidenced in a comprehensive review by Mawanda and Wallace [18]. However, studies included in these reviews also faced the same limitations in terms of sample size and cross-sectional study designs.

We aimed to summarize current evidence from longitudinal studies of the association between common clinically symptomatic bacterial infections (sepsis, lower respiratory tract infections, urinary tract infections, and skin and soft tissue infections) and risk of subsequent incident dementia or cognitive decline in adults aged 18 years and older. A secondary objective was to identify gaps in literature and recommendations for future research.

## METHODS

### Protocol and registration

We registered this systematic review with the International Prospective Register of Systematic Reviews in December 2018 (PROSPERO 2018; CRD42018119294) and published a more detailed protocol in accordance with the Preferred Reporting Items for Systematic Reviews and Meta Analyses Protocols (PRISMA) reporting guidelines [19].

### Study design

Studies eligible for inclusion were longitudinal studies such as prospective and retrospective cohort studies, secondary analyses of randomized controlled trial data, and case control studies. We included studies in which cognitive decline was measured at least 3 months following infection, to avoid associations dominated by short term, reversible cognitive impairment. Further, to assess temporality, we restricted our search to studies in which infections occurred prior to cognitive decline or dementia.

### Study population

Only studies with human participants aged 18 years and older were eligible for inclusion.

### Exposure

Exposure was defined as symptomatic illness due to common bacterial infections, either suspected clinically or confirmed by isolation of a bacterial pathogen. We identified studies investigating the following major infection types: sepsis, lower respiratory tract, skin and soft tissue, and urinary tract infections. Studies identifying specific bacterial agents alone, rather than the symptomatic disease, were excluded.

### Comparators

We only included studies in which a comparison group was present. This comparison group comprised of individuals unexposed to common bacterial infections in cohort studies and secondary analyses of longitudinal randomized controlled trial data, or a control group without dementia or cognitive decline in case control studies.

### Outcomes

Our two primary outcomes of interest were (1) incident dementia (all types) and (2) cognitive decline. We included studies in which our outcomes were defined clinically, which for dementia could be with or without neuroimaging or histopathologyresults.

### Information sources

We performed a comprehensive search across eight databases of published literature (MEDLINE, EMBASE, Global Health, PsychInfo, CINAHL Plus, Cochrane Library, Scopus, and Web of Science) and two grey literature databases (Open grey and the British Library electronic Theses Online Service) from inception to 18 March 2019. Additionally, we searched the reference lists of the included studies to identify any relevant articles not captured in our search strategy.

### Search

We searched the databases using subject headings, specific to each database, and keywords related to common bacterial infections, cognitive decline or dementia, and longitudinal study designs. These search terms were then combined using Boolean logical operators. No restrictions were placed on the language, country, or health care setting of the studies. Our search strategy was developed in consultation with a librarian at the London School of Hygiene and Tropical Medicine and was subsequently peer reviewed based on the Peer-Review for Electronic Search Strategies. We translated the final search strategy across all databases which is shown in the Supplementary Material 1.

### Study selection

The study selection process was carried out by two reviewers (RM and JAD), using the Preferred Reporting Items for Systematic Reviews and Meta-Analyses flow diagram. The two reviewers independently screened all titles, abstracts and full text articles against the eligibility criteria. A third reviewer (CWG) was consulted when there were any discrepancies.

### Data items

Data extraction was performed independently by two reviewers for all studies. We used the Population, Exposure, Comparator, Outcomes and Study characteristics framework to extract data from eligible studies (Supplementary Material 2). We pilot tested our data extraction form and modified the form accordingly. We extracted key study results, namely unadjusted and adjusted incidence effect estimates and their corresponding 95% CIs. Data on confounding variables adjusted for in each study and pre-specified sub-groups were also extracted.

### Risk of bias in individual studies

The risk of bias was assessed independently by two reviewers in line with the Cochrane collaboration approach. We classified studies as at high, medium, low or unclear risk of bias in each of the following domains: confounding, selection of participants, misclassification of variables, missing data, reverse causation, generalizability, and study power [20, 21].

### Synthesis of results

We grouped studies according to their outcome (cognitive decline or dementia) and exposure definition (common bacterial infection) and synthesized them narratively. Heterogeneity was assessed using the I squared statistic if there were two or more studies with effect estimates for the same exposure definition, outcome, and study design. Geographical location and risk of bias were explored as potential sources of heterogeneity. Other sources of heterogeneity could not be explored due to the limited data available. We also carried out sub-group analyses by age, sex, and dementia subtype where sufficient data were available.

### Quality of evidence

We assessed the overall quality of evidence for each outcome using the Grading of Recommendations, Assessment, Development and Evaluation (GRADE) tool. The following domains were assessed: study limitations, inconsistency, indirectness, imprecision, and publication bias. We rated the strength of evidence as high, moderate, low, or very low. The criteria for grading are stated in Supplementary Material 3.

## RESULTS

### Study characteristics

**Fig. 1.**
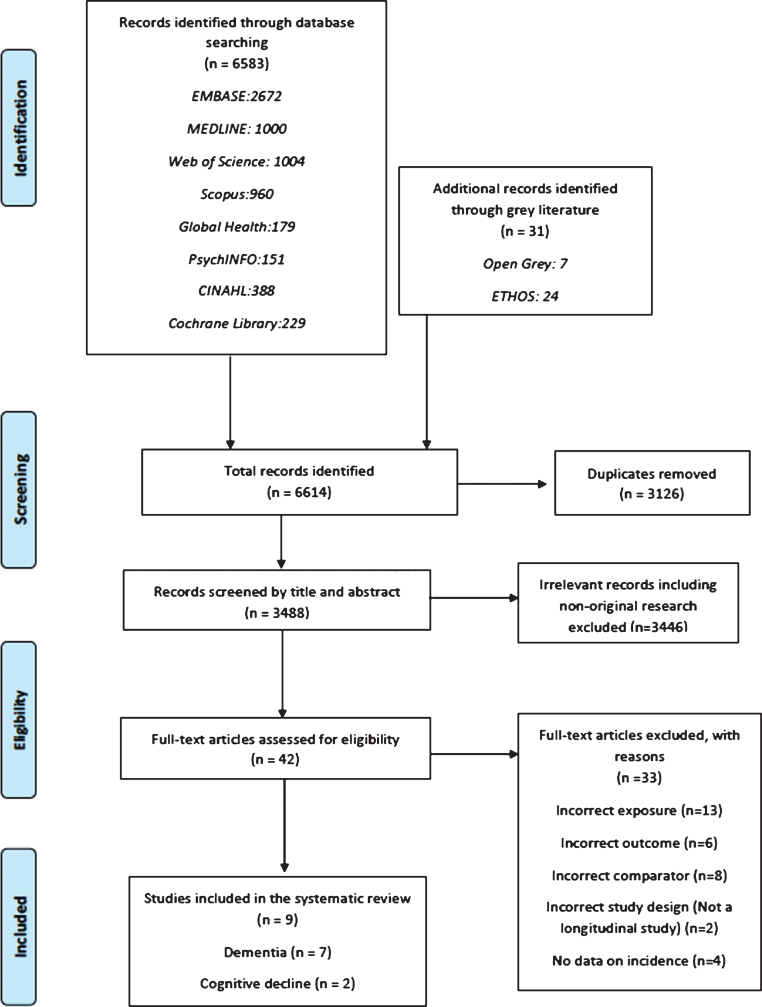
Study selection PRISMA flow diagram.

**Fig. 2.**
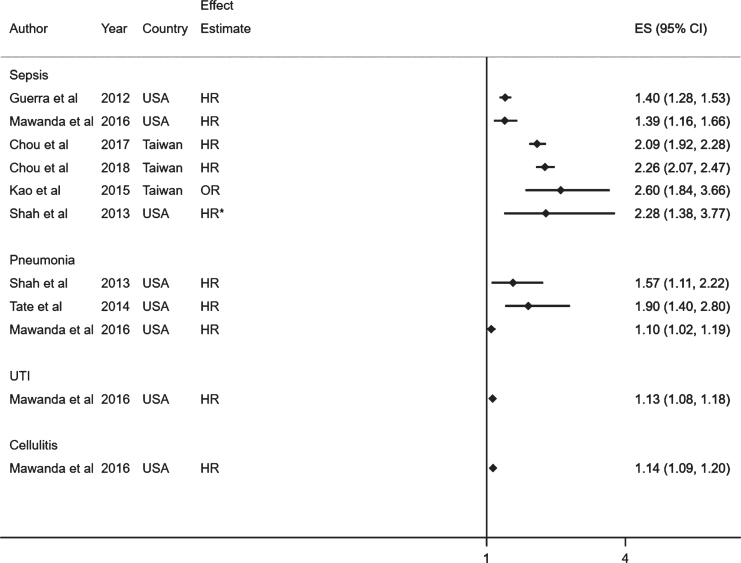
Forest plot showing the effect of infections on dementia. *Unadjusted effect estimates. The mean age (SD) in this study was 72.8 years (5.6).

**Fig. 3.**
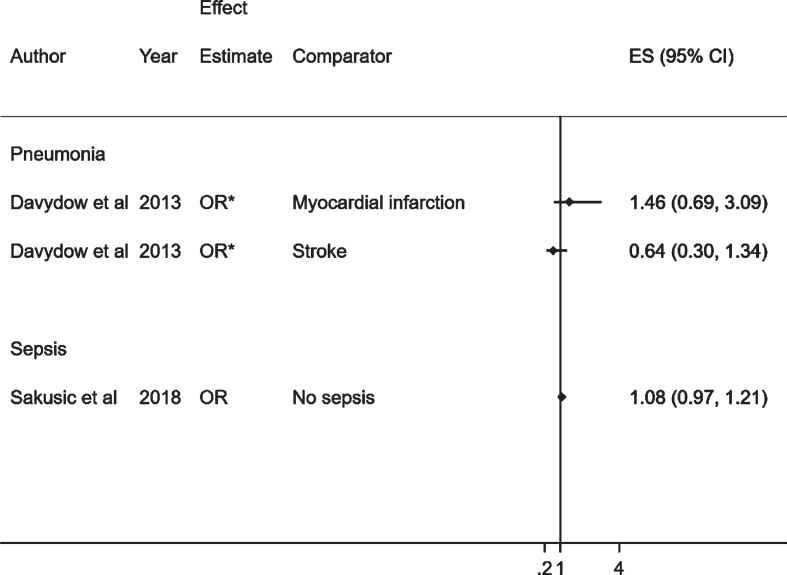
Forest plot showing the effect of common bacterial infections on cognitive decline. *Unadjusted effect estimates for the study by Davydow et al., 2013 [26]. The median age in years (SD) in this study for each exposure was as follows: pneumonia 77.1 (9.4), myocardial infarction, 75.5 (8.2) and stroke 77.0 (8.4).

In total, 3,488 studies were identified from 10 databases, after de-duplication, as outlined in Fig. 1. Of these, 42 were included in the full text screening and the reasons for exclusion were noted. Finally, 9 studies were included in the present systematic review. The study characteristics and results are summarized in Tables 1 and 2, respectively. Forest plots of the results are displayed in Figs. 2 and 3.

**Table 1:** Characteristics of studies included in the review

First Author, year of publication	Study design	Study period	Setting	Study population at recruitment	Definition and ascertainment of exposure	Definition and ascertainment of comparator	Outcome	Definition and ascertainment of	Study population characteristics (Age and male %)
**Dementia**									
**Cohort studies**									
Shah et al., 2013 [23]	Prospective cohort study	1997-unknown follow up	United States, Community dwelling adults	Adults aged 65 y or older	Pneumonia and sepsis defined using ICD-9 diagnosis codes.	Pneumonia exposure: comparators were participants never hospitalized with pneumonia. Severe sepsis exposure: comparators were participants never hospitalized with infection.	Dementia	Neuropsychiatric testing, magnetic resonance imaging evaluations and annually with the (3MS) examination.	Age 72.8 y (5.6) (mean) 42.4% male
Guerra et al., 2012 [22]	Retrospective cohort study	2005-2008	United States, Medicare beneficiaries	Adults aged 66 y and older who received intensive care and survived hospital discharge.	Diagnoses of severe sepsis assessed using a standard definition, ICD-9-CM codes.	Participants without infection	Dementia	Dementia defined using ICD-9-CM codes (290.x, 294.x, 331.x, 797.x)	Age 76.6 *y*±6.8. 48.7% male
Mawanda et al., 2016 [25]	Retrospective cohort study	2003-2012	United States, National sample of US Veterans database	Veterans aged 56 y and older during fiscal year 2003 enrolled and receiving health care at any Veterans Health Administration care facility.	Septicemia, bacteremia, pneumonia, UTI and cellulitis diagnosed using ICD-9 diagnosis codes.	Participants without a diagnosis of an extra-CNS bacterial infection	Dementia	Dementia diagnosed from using ICD-9	Age 67.7 (8.1) y (mean) and 97.9% male.
Chou et al., 2017 [29]	Retrospective cohort study	2001-2011	Taiwan, Longitudinal Health Insurance Database	Participants hospitalized with septicemia without prior dementia, age and sex matched at 1 : 2 ratio to cohort without septicemia or prior dementia.	Septicemia defined according to according to ICD-CM codes (003.1, 036.2, and 038)	Age and sex matched cohort without septicemia or prior dementia.	1. All Dementia 2. Alzheimer’s disease 3. Non-Alzheimer’s dementias	Dementia defined using ICD-9-CM codes. (290, 294.1 and 331.0)	Exposed: 65.6 y (16.8), 56% male Unexposed: Age: 65.4 y (16.7), 56% male
Chou et al. 2018 [28]	Retrospective cohort study	2001-2011	Taiwan, Longitudinal Health Insurance Database	-	Septicemia. Ascertainment not reported.	Age and sex matched cohort without septicemia or prior dementia	Vascular dementia	-	-
Tate et al., 2014 [24]	Cohort study - secondary analysis of a randomized trial	2000-2008	United States, Community dwelling adults	Adults aged 75 y and older.	ICD-9-CM codes and textual search of discharge diagnoses to identify pneumonia hospitalizations	Participants without ICD-9-CM pneumonia hospitalization codes or without pneumonia recorded in diagnoses fields	Dementia	Participants screened using 3MSE exam, ADAS-Cog scale and the clinical dementia rating.	Age=78.6 y (mean), 54% male. Exposed age = 79.5 y and 63.3% male. Unexposed Age = 78.5 y and 53.1
**Case control study**									
Kao et al. 2015 [30]	Nested case control study	2003-2011	Taiwan, Longitudinal Health Insurance Database	Adults aged 45 y and older, sex, age and year of index date matched (1 : 1) with healthy controls.	Participants hospitalized with a diagnosis of sepsis using ICD-9-CM codes within 5 y prior to the index date.	Age, sex, and year of index matched healthy controls without dementia.	Dementia	First time diagnosis of dementia using ICD-9-CM codes.	Age 75.4 (10.4 y) (mean) 44% male
**Cognitive Decline**									
**Cohort study**									
Davydow et al., 2013 [26]	Prospective cohort study	1998-2010	United States, Community dwelling adults with pneumonia, myocardial infarction and stroke hospitalizations	Adults aged over 50.	Pneumonia was diagnosed using ICD-9-CM principal diagnostic codes and to identify hospitalizations	Participants with principal discharge stroke or myocardial infarction hospitalization	Moderate to severe cognitive impairment	Cognitive impairment was assessed versions of the modified TICS interview.	Age (median) Pneumonia 77.1 (9.4), myocardial infarction, 75.5 (8.2) and stroke 77.0 (8.4)
Sakusic 2018 [27]	Nested case-control study	July 2004 - November 2015	United States, critically ill patients in ICU	Adults aged 18 y and older admitted to ICU. Excluded were those admitted to neuroscience ICU, those with cognitive impairment prior to ICU stay and those only with cognitive impairment documented within 3 months of ICU discharge.	Sepsis. Ascertainment not reported.	Cognitive impaired cases were matched to cognitively normal controls based on age, sex and having had an ICU admission	Persistent cognitive impairment	Defined as the onset of new cognitive impairment within 3-24 months after ICU discharge. Cognitive impairment identified by manually reviewing electronic health records using algorithms for cognitive impairment and dementia.	65.9 (mean age) and 54.6% male

ICD-9-CM, International Classification of Diseases, 9th revision, Clinical Modification; 3MS, Modified Mini-Mental State Examination, ADAS-Cog, Alzheimer’s Disease Assessment Scale Cognitive Subscale; ICU, Intensive Care Unit; TICS-M, Modified Telephone Interview for Cognitive Status

Of the studies included, six studies were conducted in the United States [22–27], and three in Taiwan [28–30]. Four were historical cohort studies, which used data derived from electronic health records [22, 25, 28, 29], two were prospective cohort studies [23, 26], one was a secondary analysis from a randomized controlled trial [24], and two were case-control studies [27, 30].

In total, seven studies investigated sepsis [22, 23, 25, 27–30], four assessed pneumonia [23–26], and only one study considered urinary tract infections and cellulitis [25]. Two studies investigated the effect of multiple infections on dementia [23, 25]. Infections were defined using ICD-9 (International Classification of Diseases, Ninth Revision) or ICD-9-CM (International Classification of Diseases, Ninth Revision, Clinical Modification) codes [22–26, 29, 30]. Ascertainment of infection was unclear in two studies [27, 28]. In terms of the setting in which infections were diagnosed, all studies defined infections in secondary care, with the exception of one study which included individuals receiving care at veterans health administration facilities [25]. These facilities comprise secondary care, outpatient sites and primary care settings. Two studies reported on the association between sepsis and dementia from the same study population in the Taiwanese health insurance database. Of these studies, one reported on vascular dementia as an outcome [28], while the other study reported on all types of dementia, Alzheimer’s disease, and non-Alzheimer’s disease dementia [29].

Ascertainment of dementia and cognitive decline varied across studies. Four studies defined dementia using the ICD-9 or ICD-9-CM diagnostic codes [22, 25, 29, 30], while two studies used multiple validated clinical tests to diagnose dementia [23, 24]. One study did not specify the ascertainment of dementia [28]. In one study, cognitive decline was defined using a modified version of the Telephone Interview for Cognitive Status, which is a validated measurement of cognitive impairment [26]. The other study ascertained cognitive impairment through manual review of medical records, and the use of algorithms to capture terms for cognitive impairment and dementia [27].

Sample sizes were generally smaller for studies assessing cognitive decline, ranging from 1,434 to 2,401 total population, compared to 3,602 to 417,172 in dementia studies. The age at which participants were recruited ranged widely between 18 to 75 years and older, but the mean age ranged between 65.5 to 78.6 years old.

The duration of follow up differed widely. In studies assessing cognitive decline, follow-up ranged from 3 months to 9.8 years. Among studies assessing dementia, only 3 studies reported the mean or median follow time which ranged from 2.5 to 9.0 years.

### Effect of infections on dementia

Seven studies assessed dementia as an outcome (Table 2, Fig. 2) [22–25, 28–30]. In all studies, infections were associated with an increased risk of all cause dementia or vascular dementia, with effect estimates ranging from HR 1.10 (95% CI;1.02–1.19) to OR 2.60 (95% CI;1.84–3.66).

**Table 2:** Results of studies included in the review

First Author, year of publication	Population size (N), follow-up time (y)	Subjects with outcome (or exposure for case-control studies) (N, %)	Statistical analysis method used	Main reported crude results	Main reported adjusted results	Adjusted for
**Dementia**						
**Cohort studies**						
Shah et al., 2013 [23]	5888. Dementia assessed in 3,602 participants. Followed for over 10 y.	707 (19.6%)	Cox proportional hazards regression models	**Pneumonia** HR 2.24 (95% CI; 1.62–3.11) **Severe sepsis** HR 1.98 (95% CI; 1.38–3.77)	**Pneumonia** HR 1.57 (95% CI; 1.11–2.22)	Demographics, health behaviors, other chronic health conditions, trajectories of physical and cognitive decline before pneumonia hospitalization.
Guerra et al., 2012 [22]	25,368 ICU survivors. Sepsis: 3,145, no infection: 17,151. Average follow up 2.5 *y*±0.9.	4,519 (17.8%)	Extended cox proportional hazards regression models	HR 1.63 (95% CI; 1.50,1.77)	HR 1.40 (95% CI; 1,28–1.53)	Risk factors for dementia and time dependent coefficients: Age, race, gender, cerebrovascular disease, Parkinson’s disease, alcohol abuse, hypertension, hypoglycemia and chronic renal failure.
Mawanda et al., 2016 [25]	417,172. Mean follow up 9.03 (1.1)	25,639 (6.2%)	Extended cox proportional hazards regression models	**Pneumonia** HR 1.54 (95% CI; 1.43–1.67) **Septicemia** HR 2.09 (95% CI; 1.75–2.49) **Urinary tract infection** HR 1.44 (95% CI; 1.38–1.51) **Cellulitis** HR 1.49 (95% CI; 1.42–1.56)	**Pneumonia** HR 1.10 (95% CI; 1.02–1.19) **Septicemia** HR 1.39 (95% CI; 1.16–1.66) **Urinary tract infection** HR 1.13 (95% CI; 1.08–1.18) **Cellulitis** HR 1.14 (95% CI; 1.09–1.20)	Demographic characteristics (age, gender, race/ethnicity, and annual income), medical comorbidity and psychiatric covariates (traumatic brain injury, hypertension, ischemic heart disease, cerebrovascular disease, atherosclerosis, diabetes mellitus, chronic obstructive pulmonary disease, chronic kidney disease, chronic liver disease, peptic ulcer disease/gastritis, bipolar disorder, PTSD, schizophrenia, and alcohol abuse).
Chou et al., 2017 [29]	Exposed: 20,466 Unexposed: 40,932	**Exposed:** All dementia: 832 (4.1%), Alzheimer’s disease: 46 (0.2%), non-Alzheimer’s dementias 786 (3.8%). **Unexposed:** All dementia: 1945 (4.8%), Alzheimer’s disease: 222 (0.5%) and non-Alzheimer’s dementias: 1723(4.2%)	Cox proportional hazards regression	**All dementia**: HR 1.79 (95% CI; 1.65–1.94) **Alzheimer’s disease**: HR 0.89 (95% CI;0.64–1.22) **non-Alzheimer’s dementias** 1.91 (95% CI; 1.75–2.07)	**All dementia:** HR 2.09 (95% CI; 1.92–2.28) **Alzheimer’s disease:** HR 1.15 (95% CI; 0.83–1.60) **non-Alzheimer’s dementias** 2.20 (95% CI; 2.01–2.41)	Age, sex, stroke, DM, hyperlipidemia, hypertension, depression, ARD, smoking, and NSAID use.
Chou et al. 2018 [28]	Exposed: 20,466 Unexposed: 40,932	-	Cox proportional hazards regression	HR 2.26 (95% CI; 2.07–2.47)	-	-
Tate et al., 2014 [24]	3069. Median follow up 6.1 y	523 (17.0%)	Cox proportional hazards regression models	HR 2.4 (95% CI; 1.7–3.3)	HR 1.9 (95% CI; 1.4–2.8)	Age, sex, race, site, education and baseline cognitive function.
**Case-control studies**						
Kao et al. 2015 [30]	Cases: 5,955	Cases: 122/5,955 (2.05%)	Conditional logistic regression	OR 2.68 (95% CI; 1.91–3.77)	OR 2.60 (95% CI; 1.84–3.66)	Monthly income, urbanization level, hyperlipidemia and diabetes.
	Controls: 5,955	Controls: 46/5,955 (0.77%)				
**Cognitive decline**						
**Cohort studies**						
Davydow et al., 2013 [26]	1,434 survivors. 1,711 hospitalizations; Pneumonia (*n* = 827), myocardial infarction (*n* = 450) or stroke hospitalization (*n* = 434). Follow up range (7.7–9.8 y)	Unclear	Within-person regressions	**Pneumonia versus Myocardial Infarction** OR 1.46 (95% CI; 0.69, 3.09) **Pneumonia versus Stroke** OR 0.64 (95% CI; 0.3,1.34)	-	-
**Case-control studies**						
Sakusic 2018 [27]	Cases: 2,401. Controls: 2,401. Follow up between 3-24 months	Cases: 793/2,401 (33.0%)	Conditional logistic regression	OR 1.28 (95% CI; 1.16–1.41)	OR 1.08 (95% CI; 0.97–1.21)	Charlson Comorbidity Index and N. of ICU stays.
		Controls: 736/2,401 (30.7%)				

HR, hazard ratio; PTSD, post-traumatic stress disorder; DM, diabetes mellitus; ARD, alcoholism-related disease; NSAID, non-steroidal anti-inflammatory drug; ICU, intensive care unit, OR, odds ratio.

To determine whether a meta-analysis was appropriate, we calculated heterogeneity when a sufficient number of studies were available. We decided not to meta-analyze data for the associations between sepsis or pneumonia with incident dementia due to evidence of substantial heterogeneity, I^2^ = 93.6%, *p* = <0.01 and I^2^ = 83.9%, *p* < 0.01. Due to the limited number of studies available, we could only explore geographical location and risk of bias as potential sources for heterogeneity. Heterogeneity was explored in studies assessing incident dementia following sepsis infection as shown in Supplementary Figures 1 and 2. Removing studies conducted in Taiwan reduced heterogeneity substantially (I^2^ = 0%, *p* = 0.406); however, when studies with a domain of high risk of bias were removed, heterogeneity remained high (I^2^ = 95.6%, *p* < 0.01).

### Subgroup analyses

All three studies from Taiwan reported data on subgroup analyses for age, sex, and dementia subtype as shown in Supplementary Figures 3–5. Of these, two studies reported on the association of sepsis on dementia subtype [28, 29]. In one study, sepsis was associated with an increased risk of all types of dementia 2.09 (95% CI; 1.92–2.28) and non-Alzheimer dementias 2.20 (95% CI; 2.01–2.41) [29]. However, the association of sepsis and Alzheimer’s disease HR 1.15 (95% CI; 0.83–1.60) was not statistically significant. In the other study, individuals with sepsis had an increased risk of vascular dementia HR 2.26 (95% CI; 2.07–2.47) [28]. In sub-group analysis we also explored whether the effect of infections on dementia differed by sex [29, 30]. Findings from these studies showed the association of sepsis on dementia was greater in men compared to women.

**Table 3:** Risk of bias summary assessments for individual domains

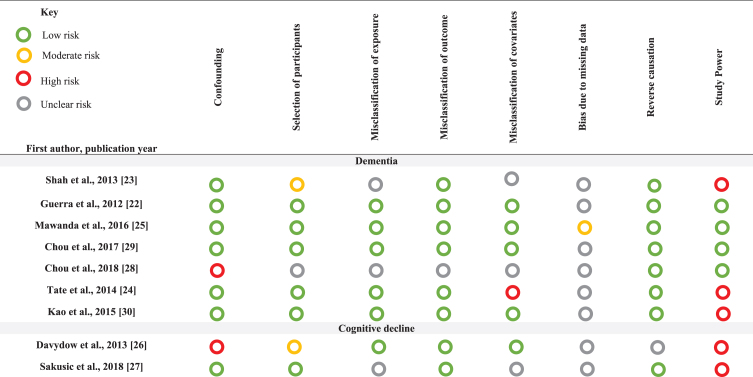

Only one study investigated the effect of age on infections and dementia [30]. Kao et al. found that compared with individuals aged 45 to 64, participants aged 65 and older showed a lower risk of dementia following sepsis HR 1.80 (95% CI; 1.65–1.97) than those aged under 45 HR 7.32 (95% CI; 1.85–28.9). However, these results are difficult to interpret due to the small number of events in the under 45 years age group (*n* = 11) compared to the 65 years and older group (*n* = 2492). This study also investigated the effect of sepsis severity on dementia and found that dementia incidence increased with mild HR 1.20 (95% CI; 1.06–1.37), moderate HR 3.37 (95% CI; 3.02–3.76) and severe HR 5.04 (95% CI; 3.98–6.37) sepsis severity. Another study also reported an increasing trend for developing dementia with increasing sepsis severity [28].

### Effect of infections on cognitive decline

Two studies assessed cognitive decline as an outcome: one following hospitalization with pneumonia and the other following admission to an intensive care unit with sepsis (Table 2, Fig. 3) [26, 27]. In one study, the effect of pneumonia hospitalization on moderate/severe cognitive impairment was compared to individuals hospitalized with stroke OR 0.64 (95% CI; 0.3–1.34) and to those with myocardial infarction OR 1.46 (95% CI; 0.69–3.09) [26]. In the other study, there was no association between sepsis and cognitive decline OR 1.08 (95% CI; 0.97–1.21) in adjusted estimates [27]. Given that the definition of infections was inconsistent in these studies, we could not pool the results into a combined effect estimate and perform a meta-analysis.

### Risk of bias

Our classifications and justifications for the risk of bias assessments are presented in Supplementary Table 1 and summarized in Table 3. Overall, none of the studies were classified as at low risk of bias across all domains. All studies assessing cognitive decline and three looking at dementia outcomes were considered at high risk of bias for study power. Studies assessing cognitive decline had particularly small sample sizes and wide confidence intervals compared to the dementia studies. The majority of studies assessing dementia scored a low risk of bias for confounding as these studies adjusted for age, sex, and other important covariates. Three studies did not have any domains at high risk of bias [22, 25, 29]. These studies all investigated the effect of sepsis on dementia, with hazard ratios ranging from 1.39 (95% CI; 1.16–1.66) to 2.09 (95% CI; 1.92–2.28). All studies were given a low rating for reverse causality as all outcomes were assessed after infection; however, three studies reported a relatively short follow up period from infection to dementia diagnosis [22, 24, 30]. Given that dementia has a long pre-clinical phase, it is thus unclear whether follow up time was long enough for dementia to develop. Further, none of the studies reported sufficient information on loss of follow up or how missing data were accounted for.

### Study quality

The overall evidence on the associations of sepsis or pneumonia with dementia were classified as of very low quality using the GRADE assessment tool. This is because these studies were rated “serious” or “very serious” for risk of bias, inconsistency, imprecision, and indirectness (Supplementary Table 2). We did not assess the overall quality of evidence for the association of other infections on dementia or cognitive decline as only a single study was available for each exposure and outcome.

## DISCUSSION

Our comprehensive systematic review identified 9 longitudinal studies examining the association of common bacterial infections with incident dementia or cognitive decline. Although a meta-analysis was not performed due to the heterogeneity of the studies, evidence from all seven studies assessing dementia found a positive association following infection with sepsis, pneumonia, urinary tract infections, or cellulitis. This association remained consistent in studies with no domains at high risk of bias. However, the overall quality of evidence was rated very low for these studies due to risk of bias, consistency, imprecision, and indirectness. Of the two studies assessing the effect of pneumonia or sepsis on cognitive decline, a lack of association was observed. However, these studies had a number of important methodological limitations including a lack of power or poor comparability, limiting the ability to draw accurate conclusions from the findings.

### Heterogeneity

The high heterogeneity observed between the studies assessing incident dementia precluded a meta-analysis, despite the studies being homogenous in terms of exposure, outcome, and study design. A major source of heterogeneity may have been the differences in the country in which the study was conducted. Studies assessing the effect of sepsis on dementia were either from the United States or Taiwan, with studies from Taiwan reporting much greater effect estimates compared to those from the United States. When studies from Taiwan were removed from the meta-analysis, heterogeneity reduced substantially. However, due to the small number of studies available, we were unable to quantitatively explore heterogeneity in studies from Taiwan. Studies assessing pneumonia were all conducted in the United States, but also had high heterogeneity, however, the paucity of studies limited the ability to quantitatively explore sources of the heterogeneity.

There are a number of possible explanations for the substantial heterogeneity observed. Varying assessments were used to diagnose dementia including neuropsychological tests, ICD-9 or ICD-9-CM codes, and magnetic resonance imaging. Studies using electronic health records rely on routine medical diagnoses which can result in misclassification given that dementia is frequently under-diagnosed in these databases [31, 32]. However, further evidence suggests that recording of dementia diagnoses is changing over time, with improvements observed in more recent years [33, 34]. Another potential issue arising from routine healthcare data is that individuals with illnesses encounter health services more frequently compared to healthy people, which could increase the likelihood of getting a dementia diagnosis. This may, however, be more likely to occur among those with chronic illnesses requiring ongoing management than with acute infections.

Another source of heterogeneity could have come from differences in the adjustment of confounders, given that the study that adjusted for a wide range of confounders including demographics, psychiatric and medical comorbidity reported weaker effect estimates in comparison to the other studies [25]. Additionally, differences in the age at recruitment and mean age of the study populations may also account for the heterogeneity. This is of importance as the risk of developing infections increases with age [35] and in turn older adults have a greater chance of developing dementia, with the risk doubling every 5 years after the age of 65 [36]. Additionally, sex representation, which ranged from 44% to 97% for men, may have contributed to heterogeneity. In our subgroup analyses, we observed differences in sex in the Taiwanese studies looking at the association between sepsis and dementia, with men at a greater risk of dementia compared to women [29, 30]. Studies from Europe and the United States suggest that there is gender variation in the reduction of age-specific dementia, with some reporting a greater decline in men [3, 6, 9] and others in women [5, 8].

One study did not find an association between sepsis and Alzheimer’s disease. The study suggested a reason for this may be due to the low prevalence for a causative pathogen of Alzheimer’s disease, *B. burgdorferi*, in Taiwan [29]. Another reason could be the potential to misdiagnose Alzheimer’s disease. A systematic review investigating the validation of dementia cases in routine health care data from Europe, North America, and Australia found that positive predictive values of Alzheimer’s disease ranged from 57% to 100% [37].

### Cognitive decline

Evidence for any association of common bacterial infections with cognitive decline was limited. The study by Davydow et al. faced a number of limitations. First, there was no adjustment for confounders in this study [26]. Second, individuals hospitalized with pneumonia were compared to those with stroke and myocardial infarction. This raises issues on the suitability of these comparator groups as stroke and myocardial infarction are both risk factors for dementia and may increase the risk of pneumonia [38, 39]. The authors stated that these analyses were based on a hypothetical population, and as such the results may not be generalizable to a particular group of people. Taken together, these limitations make it difficult to extrapolate any meaningful conclusions from these results.

### Comparison with previous studies

Although the association between common bacterial infections and cognitive decline was unclear, evidence from previous longitudinal studies suggests that individuals with bacterial infections are associated with worsening cognitive impairment [16, 40], with one study showing a decline in the mean score of cognitive ability, assessed using the Danish intelligence test, following hospitalization with sepsis, skin, respiratory and urological infections [41]. Other studies have found a link between sepsis and specific cognitive domains after long-term follow up [42].

Regarding the link between bacterial infections and dementia, our findings are consistent with evidence from a nested-case control study using UK primary care data which suggested that episodes of infections were associated with an increased likelihood of a dementia diagnosis [43]. This study assessed the overall effect of infections on dementia, including urinary tract and skin infections, rather than the individual effect of each infection on dementia. Because of this, the study was not eligible for inclusion.

Individual bacterial pathogens including *Helicobacter pylori*, *Chlamydia pneumoniae, Borelia burgdorferi*, and oral *spirochetal Trepenoma* have been linked to Alzheimer’s disease, primarily in serology based and post mortem brain studies [10, 18]. *Chlamydia pneumonia* and *spirochetes* are the focus of previous reviews and have been frequently associated with Alzheimer’s disease [10, 11, 44–46]. In their review, Mawanda and Wallace suggested chronic bacterial infections, such as tuberculosis, are associated with amyloid deposition, a key hallmark of Alzheimer’s disease. This is further supported in a study by Emery et al., which demonstrated an increase in *actinobacteria* in the brains of individuals with Alzheimer’s disease compared to controls [47]. However, no single microorganism has been identified as the sole pathogen responsible for Alzheimer’s disease.

### Mechanisms

Mechanisms underlying the association between infections and subsequent dementia are unclear [48, 49], but several plausible pathophysiological pathways have been proposed. One such potential pathway is through systemic inflammation. Infections can induce systemic inflammation through the release of pro-inflammatory mediators which can cross the blood-brain barrier and activate cytotoxic microglia. This may result in a deterioration of cognitive function and thus increasing the risk of developing dementia [10]. In support of this mechanism, evidence from a growing number of longitudinal studies suggests that markers of systematic inflammation, such as tumor necrosis factor, nitric oxide synthase, and interleukin IL-1β, IL-6, and IL-18, are involved in the pathogenesis of dementia [50, 51]. Recent findings demonstrate that when sepsis is induced in animal models, it triggers systemic inflammation which leads to accumulation of amyloid-β and cognitive dysfunction [52, 53].

Alternatively, it is also possible that the association between infections and dementia is non-causal and may be a result of the co-occurrence of age-related pathologies. The immune system deteriorates with age, increasing incidence of infection. Conversely, the aging immune system also induces a chronic inflammatory state which leads to tissue damage and inflammatory disease and accelerates age-related diseases such as Alzheimer’s disease [54, 55]. Nevertheless, the present review focused only on longitudinal studies which provide evidence of appropriate temporality between infections and dementia, thus adding to the likelihood of a possible causal relationship.

### Strengths and limitations

Strengths of our study include a comprehensive search using multiple databases of published and grey literature, with no restrictions on the date, language or geographical location of the studies. Our search strategy was detailed and peer reviewed. We registered and published our protocol in order to increase the transparency of our findings. Other strengths include the inclusion of longitudinal studies to minimize reverse causality, a minimum 3 month follow up period to avoid capturing short term cognitive impairment, and requiring the use of a comparator group without infections in order to provide evidence on causality.

There are several limitations to this systematic review. First, there was a small number of longitudinal studies available, particularly for cognitive decline outcomes. Second, the high heterogeneity between the studies meant that it was not feasible to perform a meta-analysis. Third, given the long pre-clinical phase of dementia and the evidence that individuals with dementia are at a greater risk of hospitalizations and common bacterial infections [56], we cannot rule out the possibility of reverse causality. Fourth, these studies did not account for past hospitalizations with infections, as such we cannot rule out the effect of previous infections on the risk of dementia. Fifth, the generalizability of these studies is of concern. All studies were conducted in the United States or Taiwan, and there were no studies from Europe or low- or middle-income countries. Further to this, infections were predominantly diagnosed in a hospital setting. These findings may thus not be representative of individuals with less severe infections that did not require hospitalization. This may have led to an underestimation of people with infections.

### Implications for research and practice

The paucity of studies available highlights the need for further large scale, longitudinal studies from populations across the world.

Our sub-group analyses suggested that the severity of sepsis is associated with an increased risk of dementia. Further research on the effect of severity, frequency, and timing of infections on cognitive decline and dementia is warranted. This will be important for identifying the sub-populations most at risk of dementia. In line with this rationale, previous studies have identified a dose-response relationship between hospital contacts with infection and cognitive ability [41]. Additionally, there is evidence of gender variation between infections and dementia, as such, more work is needed to explore this possible link further as it may have implications on prevention strategies in men and women.

A key drawback of the studies included was the fact that infections were predominantly diagnosed in secondary care. This is an issue as hospitalization itself has been associated with incident cognitive decline and dementia [57–59]. Hospitalized patients are at a greater risk of nosocomial infections [60], delirium [61], and functional decline [62], which may also increase the risk of dementia. Therefore, individuals hospitalized with infections may not be representative of those with infections diagnosed in primary care. In future, studies could investigate the effect of infections diagnosed in different health care settings. Additionally, the bacterial agents responsible for infections acquired in the community or in hospital settings differ, and as such it is possible that these pathogens could have differing effects on dementia, if any at all. Future longitudinal studies investigating the link between laboratory confirmed bacterial agents and dementia could shed further light on causality.

Research on infections as potential risk factors for dementia faces a number of challenges. Firstly, the etiology of dementia is multifactorial and is likely to involve an interplay of genetic, environmental, and lifestyle factors. In addition, the fact that age is the single greatest risk factor for dementia raises challenges in disentangling the pathophysiological effects of age on dementia with the independent effects of infections on dementia. Secondly, the pathophysiological processes of dementia may begin years before dementia is diagnosed, and as such it is possible that the preclinical phase of dementia may be underway before infection occurs. Future studies with a follow up time sufficient enough for dementia to develop are recommended in order to minimize the possibility of reverse causality. Moreover, given that infections may trigger delirium, it is important for future studies to ensure that individuals are followed up long enough for delirium to resolve in order to help distinguish between delirium and long-term cognitive decline. However, as delirium itself is associated with cognitive decline and dementia, there is a need to better understand whether the pathological processes of infections on long-term cognitive decline are independent of delirium. Third, prospective cohort studies on infections and dementia are susceptible to selection bias. Individuals with more severe infections are associated with attrition as they are more likely to experience greater morbidity and an increased risk of mortality compared to those with less severe infections. Additionally, cognitive decline and dementia are associated with attrition during follow up and drop-out due to death [63, 64], which may underestimate the true effect of infections on dementia. Further studies could tackle this limitation by performing an analysis of attrition to investigate whether those lost to follow up were more likely to have impaired cognitive function. Loss of follow up is minimized in routine healthcare datasets; however, one of the limitations of these datasets is that they rely on individuals seeking health care services. As a result, health seeking behavior could affect the likelihood of a dementia diagnosis. Future studies should consider accounting for health-seeking behavior in their design oranalysis.

This review suggests that infections may be involved in the development of dementia. These findings could have clinical implications in the early recognition and treatment of infections, particularly in the older population who are more susceptible to infections and are at a greater risk of dementia. Additionally, other implications include the need for strategies to improve infection control and to identify sub-populations at risk of infections and dementia.

### Conclusions

Our systematic review suggests that sepsis, pneumonia, urinary tract infections, and cellulitis may be associated with an increased risk of dementia, after adjustment for multiple confounders. However, due to the paucity of longitudinal studies, further evidence from high quality studies is needed to confirm this association. Given that evidence on cognitive decline was limited by a lack of studies and small sample sizes, further large scale, well-powered studies are needed to investigate the effect of infections on cognitive decline. Infections are well-recognized to trigger delirium, as such, it is important for future work to distinguish whether the potential association between infections and cognitive decline is independent of delirium. Common bacterial infections frequently occur in the elderly, who are at an increased risk of dementia, and thus a better understanding of their role in dementia development could inform dementia risk reduction strategies.

## Supplementary Material

Supplementary Material

Supplementary Table
