# Supplementary material for: Common Bacterial Infections and Risk of Dementia or Cognitive Decline: A Systematic Review
Source: J Alzheimers Dis. 2020 Aug 18;76(4):1609–26. doi: 10.3233/JAD-200303 (PMC7504996; doi:10.3233/JAD-200303)

SUPPLEMENTARY MATERIAL

Common Bacterial Infections and Risk of Dementia or Cognitive Decline: A Systematic Review

Supplementary Material 1: Search Strategy

a) MEDLINE (OVID) search strategy

1. Pneumonia/ or pneumonia, bacterial/
2. Pneumonia.ti,ab
3. Lower respiratory tract infection*.ti,ab
4. (LRTI or LRTIS).ti,ab.
5. Exp urinary tract infections/
6. (Urinary adj5 infection*).ti,ab.
7. (UTI or UTIS).ti,ab
8. exp Cystitis/
9. (bacteriuria or pyuria or cystitis or pyelonephritis or cellulitis).ti,ab.
10. exp cellulitis/
11. (Skin and soft tissue infection).mp.
12. exp sepsis/
13. (septic* or sepsis or septic?emia or systematic inflammatory response syndrome or blood stream infection or py?emia).ti,ab.
14. 1 or 2 or 3 or 4 or 5 or 6 or 7 or 8 or 9 or 10 or 11 or 12 or 13
15. Exp dementia/
16. Exp prion diseases/
17. (huntington* or kluver-bucy or prion disease or Creutzfeldt-jakob or primary progressive aphasia).ti,ab
18. (Dement* or Alzheimer*).ti,ab
19. (Lewy*adj2 bod*).ti,ab.
20. Cognitive dysfunction/
21. (Mild cognitive impairment or MCI).ti,ab
22. ((cognit* or memory or cerebr* or mental*) adj3 (declin* or impair* or los* or deteriorat* or degenerat* or complain* or disturb* or disorder*)).ti,ab.
23. Cognitive function.ti,ab.
24. 13 or 14 or 15 or 16 or 17 or 18 or 19 or 20 or 21
25. cohort studies/ or longitudinal study/ or follow-up study/ or prospective study/ or retrospective study/ or cohort.ti,ab. or longitudinal.ti,ab. or prospective.ti,ab. or retrospective.ti,ab.
26. Case-Control Studies/ or Control Groups/ or Matched-Pair Analysis/ or ((case* adj5 control*) or (case adj3 comparison*) or control group*).ti,ab.
27. Incidence/ or incidence.ti,ab,kw.
28. (hazard ratio or HR or odds ratio or relative risk or RR).ti,ab.
29. 23 or 24 or 25 or 26
30. 14 and 24 and 29

b) Embase (OVID) search strategy

1. Bacterial pneumonia/ or Pneumonia/
2. Pneumonia.ti,ab
3. Lower respiratory tract infection*.ti,ab
4. (LRTI or LRTIS).ti,ab.
5. Exp Urinary tract infections/
6. exp Bacteriuria/
7. exp Pyuria/
8. (Urinary adj5 infection*).ti,ab.
9. (UTI or UTIS).ti,ab
10. Exp cystitis/
11. (bacteriuria or pyuria or cystitis or pyelonephritis or cellulitis).ti,ab.
12. exp cellulitis/
13. (Skin and soft tissue infection).mp.
14. exp sepsis/
15. (septic* or sepsis or septic?emia or systematic inflammatory response syndrome or blood stream infection or py?emia).ti,ab.
16. 1 or 2 or 3 or 4 or 5 or 6 or 7 or 8 or 9 or 10 or 11 or 12 or 13 or 14 or 15
17. Exp dementia
18. Exp Creutzfeldt-jakob disease
19. (huntington* or kluver-bucy or prion disease or Creutzfeldt-jakob or primary progressive aphasia).ti,ab
20. Dement*.ti,ab.
21. Exp mild cognitive impairment/
22. (Mild cognitive impairment or MCI)ti,ab
23. ((cognit* or memory or cerebr* or mental*) adj3 (declin* or impair* or los* or deteriorat* or degenerat* or complain* or disturb* or disorder*)).ti,ab.
24. Cognitive function.ti,ab.
25. Alzheimer*.ti,ab
26. (Lewy*adj2 bod*).ti,ab.
27. 15 or 16 or 17 or 18 or 19 or 20 or 21 or 22 or 23 or 24
28. cohort analysis/ or longitudinal study/ or follow-up/ or prospective study/ or retrospective studies/ or cohort.ti,ab. or longitudinal.ti,ab. or prospective.ti,ab. or retrospective.ti,ab.
29. Case control study/ or Control Group/ or ((case* adj5 control*) or (case adj3 comparison*) or control group*).ti,ab.
30. Incidence/ or incidence.ti,ab,kw.
31. (hazard ratio or HR or odds ratio or relative risk or RR).ti,ab.
32. 26 or 27 or 28 or 29
33. 14 and 25 and 30

c) Global health (OVID) search strategy

1. Pneumonia/ or bacterial pneumonia/
2. Pneumonia.ti,ab
3. Lower respiratory tract infection*.ti,ab
4. Lower respiratory tract infections/
5. (LRTI or LRTIS).ti,ab.

6. Exp Urinary tract infections/
7. Exp Bacteriuria/
8. (Urinary adj5 infection*).ti,ab.
9. (UTI or UTIS).ti,ab
10. Exp cystitis/
11. (bacteriuria or pyuria or cystitis or pyelonephritis or cellulitis).ti,ab.
12. exp cellulitis/
13. (Skin and soft tissue infection).mp.
14. Exp sepsis/
15. (septic* or sepsis or septic?emia or systematic inflammatory response syndrome or blood stream infection or py?emia).ti,ab.
16. 1 or 2 or 3 or 4 or 5 or 6 or 7 or 8 or 9 or 10 or 11 or 12 or 13 or 14 or 15
17. Exp dementia/
18. Exp creutzfeldt-jakob disease/or exp prion diseases/
19. (Dement* or Alzheimer*).ti,ab
20. (huntington* or kluver-bucy or prion disease or Creutzfeldt-jakob or primary progressive aphasia).ti,ab
21. (Mild cognitive impairment or MCI).ti,ab.
22. Cognitive function.ti,ab.
23. ((cognit* or memory or cerebr* or mental*) adj3 (declin* or impair* or los* or deteriorat* or degenerat* or complain* or disturb* or disorder*)).ti,ab.
24. (Lewy*adj2 bod*).ti,ab.
25. 15 or 16 or 17 or 18 or 19 or 20 or 21 or 22 or 23 or 24
26. cohort studies/ or longitudinal studies/ or follow up/ or retrospective studies/ or cohort.ti,ab. or longitudinal.ti,ab. or prospective.ti,ab. or retrospective.ti,ab.
27. Case-Control Studies/ or ((case* adj5 control*) or (case adj3 comparison*) or control group*).ti,ab.
28. Incidence/ or incidence.ti,ab,kw.
29. (hazard ratio or HR or odds ratio or relative risk or RR).ti,ab.
30. 26 or 27 or 28 or 29
31. 16 and 25 and 30

d) PsychINFO (OVID) search strategy

1. Pneumonia/
2. Pneumonia.ti,ab
3. Lower respiratory tract infection*.ti,ab
4. (LRTI or LRTIS).ti,ab.
5. (Urinary adj5 infection*).ti,ab.
6. (UTI or UTIS).ti,ab
7. (bacteriuria or pyuria or cystitis or pyelonephritis or cellulitis).ti,ab.
8. (Skin and soft tissue infection).mp.
9. (septic* or sepsis or septic?emia or systematic inflammatory response syndrome or blood stream infection or py?emia).ti,ab.
10. 1 or 2 or 3 or 4 or 5 or 6 or 7 or 8 or 9
11. Exp dementia
12. Exp kluver bucy syndrome/

13. Exp huntingtons disease/
14. (huntington* or kluver-bucy or prion disease or Creutzfeldt-jakob or primary progressive aphasia).ti,ab
15. (Dement* or Alzheimer*).ti,ab
16. cognitive impairment/
17. (Mild cognitive impairment or MCI)ti,ab
18. Cognitive function.ti,ab.
19. ((cognit* or memory or cerebr* or mental*) adj3 (declin* or impair* or los* or deteriorat* or degenerat* or complain* or disturb* or disorder*)).ti,ab.
20. (Lewy*adj2 bod*).ti,ab.
21. 11 or 12 or 13 or 14 or 15 or 16 or 17 or 18 or 19 or 20
22. cohort analysis/ or longitudinal studies/ or followup studies/ or prospective studies/ or retrospective studies/ or cohort.ti,ab. or longitudinal.ti,ab. or prospective.ti,ab. or retrospective.ti,ab.
23. ((case* adj5 control*) or (case adj3 comparison*) or control group*).ti,ab.
24. Incidence/ or incidence.ti,ab,kw.
25. (hazard ratio or HR or odds ratio or relative risk or RR).ti,ab.
26. 22 or 23 or 24 or 25
27. 10 and 21 and 26

e) CINAHL Plus search strategy

S1. (MH “pneumonia, bacterial”)
S2. (MH “Pneumonia”)
S3. TI “pneumonia” or AB “pneumonia”
S4. TI “lower respiratory tract infection*” or AB “lower respiratory tract infection”
S5. TI LRTI or AB LRTI or TI LRTIs or AB LRTIs
S6. (MH “urinary tract infections+”)
S7. TI “urinary tract infection*” or AB “urinary tract infection*”
S8. TI “UTI” or AB “UTI” or TI “UTIs” or AB “UTIs”
S9. (MH “cystitis+”)
S10. TI “cystitis” or AB “cystitis”
S11. TI “bacteriuria” or AB “bacteriuria”
S12. TI “pyuria” or AB “pyuria”
S13. (MH “pyelonephritis”)
S14. TI “pyelonephritis” or AB “pyelonephritis”
S15. (MH “cellulitis+”)
S16. TI “cellulitis” or AB “cellulitis”
S17. (TI “skin and soft tissue infection” or AB “skin and soft tissue infection”
S18. TI "septic*" or AB "septic*" or TI sepsis or AB sepsis or TI septic#emia or AB septic#emia or TI "systematic inflammatory response syndrome" or AB "systematic inflammatory response syndrome" or TI "blood stream infection" or AB "blood stream infection" or TI py#emia or AB py#emia
S19. (MH "Sepsis+") OR (MH "Systemic Inflammatory Response Syndrome+") OR (MH "Shock, Septic+")
S20. S1 or S2 or S3 or S4 or S5 or S6 or S7 or S8 or S9 or S10 or S11 or S12 or S13 or S14 or S15 or S16 or S17 or S18 or S19

S21. (MH “Dementia+”) or (MH “Frontotemporal Dementia+”) or (MH “Dementia, Vascular+” or (MH “Dementia, Multi-Infarct+” or (MH “Lewy Body Disease”) or (MH “Dementia, senile+”) or (MH “Dementia, presenile+”)
S22. (MH”Creutzfeldt-Jakob syndrome+) or (MH “prion diseases+”)
S23. (MH “Alzheimer’s Disease”)
S24. (MH “Huntington’s Disease”)
S25. (MH “Pick Disease of the Brain”)
S26. TI “Dement*” or AB “Dement*”
S27. TI “Alzheimer*” or AB “Alzheimer*”
S28. TI “huntington*” or AB “huntington*” or TI “kluver-bucy” or AB “kluver-bucy” or TI “Creutzfeldt-Jakob” or AB “Creutzfeldt-Jakob” or TI “primary progressive aphasia” or AB “primary progressive aphasia”
S29. TI “Mild cognitive impairment” or AB “Mild cognitive impairment” or TI “MCI” or AB “MCI”
S30. TI “Cognitive function” or AB “cognitive function”
S31. TI ((cognit* or memory or cerebr* or mental*) N3 (declin* or impair* or los* or deteriorat* or degenerat* or complain* or disturb* or disorder*)) OR AB ((cognit* or memory or cerebr* or mental*) N3 (declin* or impair* or los* or deteriorat* or degenerat* or complain* or disturb* or disorder*))
S32. TI “Lewy*bod*” or AB “Lewy* bod*”
S33. S21 or S22 or S23 or S24 or S25 or S26 or S27 or S28 or S29 or S30 or S31 or S32
S34. (MH “Prospective studies”)
S35. (MH “Retrospective studies”)
S36. TI “cohort” or AB “cohort” or TI “longitudinal” or AB “longitudinal” or TI “prospective” or AB “prospective” or TI “retrospective” or AB “retrospective”
S37. (MH “Case Control Studies”)
S38. (MH “Control Group”)
S39. (MH “Matched-Pair Analysis”)
S40. TI “case* control* or AB “case* control*” or TI “case comparison*” or AB “case comparison*” or TI “control group*” or AB “control group*”
S41. (MH “incidence”)
S42. Incidence
S43. TI “Hazard Ratio” or AB “Hazard Ratio” or TI HR or AB HR or TI “odds ratio” or AB “odds Ratio” or TI “relative risk” or AB “relative risk” or TI RR or AB RR
S44. S34 or S35 or S36 or S37 or S38 or S39 or S40 or S41 or S42 or S43
S45. S20 and S33 and S44

f) Cochrane Library search strategy

1. MESH Descriptor: [bacterial infections] this term only
2. Bacterial infection:ti,ab,kw
3. Pneumonia:ti,ab,kw
4. MeSH descriptor [Pneumonia] this term only
5. MeSH descriptor [Pneumonia, bacterial] explode all trees
6. (Lower respiratory tract infection*):ti,ab,kw
7. (LRTI or LRTIS):ti,ab,kw
8. MeSH descriptor: [Urinary tract infections] explode all trees

9. (Urinary tract infections):ti,ab,kw
10. (UTI or UTIS):ti,ab,kw
11. MeSH descriptor: [Bacteriuria] explode all trees
12. MeSH descriptor: [Pyuria] explode all trees
13. (Bacteriuria or pyuria or pyelonephritis or cystitis or cellulitis):ti,ab,kw
14. MeSH descriptor: [cystitis] explode all trees
15. MeSH descriptor: [cellulitis] explode all trees
16. Skin and soft tissue infection:ti,ab,kw
17. MeSH descriptor: [sepsis] explode all trees
18. (septic* or sepsis or septic?emia or systematic inflammatory response syndrome or blood stream infection or py?emia).ti,ab,kw
19. #1 or #2 or #3 or #4 or #5 or #6 or #7 or# 8 or #9 or #10 or #11 or #12 or #13 or #14 or #15 or #16 or #17 or #18
20. MeSH descriptor: [dementia] explode all trees
21. MeSH descriptor: [Alzheimer disease] explode all trees
22. (Dement*):ti,ab,kw
23. (Alzheimer*):ti,ab,kw
24. MeSH descriptor: [Dementia, multi-infarct] explode all trees
25. MeSH descriptor: [prion diseases] explode all trees
26. MeSH descriptor: [pick disease of the brain] explode all trees
27. (Mild cognitive impairment or MCI):ti,ab,kw
28. (Cognitive function):ti,ab,kw
29. MeSH descriptor: [cognitive dysfunction] explode all trees
30. (Lewy* bod*):ti,ab,kw
31. #20 or #21 or #22 or #23 or #24 or #25 or #26 or #27 or #28 or #29 or #30
32. MeSH descriptor: [cohort studies] explode all trees
33. MeSH descriptor: [longitudinal] explode all trees
34. (cohort or longitudinal or prospective or retrospective):ti,ab,kw
35. MeSH descriptor: [case-control groups] explode all trees
36. MeSH descriptor: [matched-pair analysis] explode all trees
37. MeSH descriptor: [control groups] explode all trees
38. Case-Control Studies/ or ((case* control*) or (case comparison*) or control group*).ti,ab,kw
39. MeSH descriptor: [incidence] explode all trees
40. (hazard ratio or HR or odds ratio or relative risk or RR).ti,ab.
41. #32 or #33 or #34 or #35 or #36 or #37 or #38 or #39 or #40
42. #17 and #30 and #40

g) Web of Science search strategy

1. TS=(pneumonia or “lower respiratory tract infection” or LRTI OR LRTIs or “urinary tract infection*” or UTI or UTIs or bacteriuria or pyuria or pyelonephritis or cystitis or “skin and soft tissue infection” or "septic*" or sepsis or septic?emia or "systematic inflammatory response syndrome" or "blood stream infection" or py?emia)

2. TS=(dement* or Alzheimer* or “lewy* bod*” or “primary progressive aphasia” or huntington* or “kluver-bucy” or “prion disease” or “Creutzfeldt-jakob disease” or “mild

cognitive impairment” or MCI or “cognitive function”) OR TS=((cognit* or memory or cerebr* or mental*) NEAR/3 (declin* or impair* or los* or deteriorat* or degenerat* or complain* or disturb* or disorder*))

3. TS=(cohort study or longitudinal or “follow up” or prospective or retrospective or “case control” or “case comparison” or “control group” or incidence or “hazard ratio” or HR or “odds ratio” or “relative risk” or RR)

4 #1 and #2 and #3

h) Scopus search strategy
((TITLE-ABS (pneumonia OR "lower respiratory tract infection*" OR lrti OR lrtis OR "urinary tract infection*" OR uti OR utis OR bacteriuria OR pyuria OR pyelonephritis OR cystitis OR "skin and soft tissue infection" OR cellulitis OR sepsis OR "septic*" OR "sepsis" OR septic?emia OR "systematic inflammatory response syndrome" OR "blood stream infection" OR py?emia))) AND (((TITLE-ABS (dement* OR alzheimer* OR "lewy* bod*" OR "primary progressive aphasia" OR "huntington*" OR "kluver-bucy" OR "creutzfeldt-jakob" OR "mild cognitive impairment" OR mci OR "cognitive function"))) OR ((TITLE-ABS (cognit* OR memory OR cerebr* OR mental*) W/3 (declin* OR impair* OR los* OR deteriorat* OR degenerat* OR complain* OR disturb* OR disorder*))))) AND ((TITLE-ABS (cohort OR longitudinal OR "follow up" OR prospective OR retrospective OR "case control" OR "control group" OR "incidence" OR "hazard ratio" OR hr OR "odds ratio" OR "relative risk" OR rr)))

i) Open Grey search strategy
1. “Bacteria* infection*” and dement*
2. “Bacteria* infection*” and Alzheimer*
3. “Bacteria* infection” and cognit*
4. Pneumonia and dement*
5. Pneumonia and Alzheimer*
6. Pneumonia and cognit*
7. “Lower respiratory tract infection*” and dement*
8. “Lower respiratory tract infection*” and Alzheimer*
9. “Lower respiratory tract infection*” and cognit*
10. “Urinary tract infection” and dement*
11. “Urinary tract infection” and Alzheimer*
12. “Urinary tract infection” and cognit*
13. cystitis and dement*
14. cystitis and Alzheimer*
15. Cystitis and cognit*
16. Bacteriuria and dement*
17. Bacteriuria and Alzheimer*
18. Bacteriuria and cognit*
19. Pyelonephritis and dement*

20. Pyelonephritis and Alzheimer*
21. Pyelonephritis and cognit*
22. cellulitis and dement*
23. cellulitis and Alzheimer*
24. cellulitis and cognit*
25. “skin and soft tissue infection” and dement*
26. “skin and soft tissue infection” and Alzheimer*
27. “skin and soft tissue infection” and cognit*
28. Sepsis and dement*
29. Sepsis and Alzheimer*
30. Sepsis and Cognit*
31. Septic* and dement*
32. Septic* and Alzheimer*
33. Septic* and cognit*

j) British Library of Electronic Theses (EThOS) search strategy

1. Bacterial infection and dementia
2. Bacterial infection and Alzheimer
3. Bacterial infection and cognition
4. Pneumonia and dementia
5. Pneumonia and Alzheimer
6. Pneumonia and cognition
7. Lower respiratory tract infection and dementia
8. Lower respiratory tract infection and Alzheimer
9. Lower respiratory tract infection and cognition
10. Urinary tract infection and dementia
11. Urinary tract infection and Alzheimer
12. Urinary tract infection and cognition
13. Cystitis and dementia
14. Cystitis and Alzheimer
15. Cystitis and cognition
16. Bacteriuria and dementia
17. Bacteriuria and Alzheimer
18. Bacteriuria and cognition
19. Pyelonephritis and dement*
20. Pyelonephritis and Alzheimer*
21. Pyelonephritis and cognit*
22. cellulitis and dementia
23. cellulitis and Alzheimer
24. cellulitis and cognition
25. skin and soft tissue infection and dementia
26. “skin and soft tissue infection” and Alzheimer*
27. “skin and soft tissue infection” and cognit*

28. Sepsis and dementia
29. Sepsis and alzheimer
30. Sepsis and cognition
31. Septicemia and dementia
32. Septicemia and Alzheimer
33. Septicemia and cognition
34. Septicaemia and dementia
35. Septicaemia and Alzheimer
36. Septicaemia and cognition

Supplementary Material 2: Extracted Data Items

Population: age (mean, median or range), sex, inclusion and exclusion criteria.
Exposure: definition of exposure, type of bacterial infection, cause of sepsis, number of exposed.
Comparators: identification and definition of comparator, number of comparators.
Outcomes: definition of outcome and identification of cognitive decline and dementia, number of participants with the outcome.
Study characteristics: authors, name of study, year of publication, study design, type of longitudinal study, healthcare setting, country, sample size, duration of follow-up.

Supplementary Material 3: Grade Quality Assessment Reasons to Up- Or Downgrade

1. Risk of bias:

- Not serious if >50% of studies have no domain which is at high risk of bias.
- Serious if studies are judged to be between 'not serious' and 'very serious'.
- Very serious if studies which have two or more domains at high risk of bias represent more than 50% of the total studies and contribute more than 50% to any meta-analyses.

2. Inconsistency:

- Not serious if have 0, serious if have 1, and very serious if have two or more of the following:
- Heterogeneity is moderate (I^2 ~30-60%), or above.
- Wide variance of point estimates across different studies.
- Minimal overlap of confidence intervals.

3. Indirectness:

Not serious if have 0, serious if have 1, and very serious if have two of the following:

- An indirect comparison (for example study A compares to a group without any infection and study B compares to a group without a specific infection category).
- Studies differ in terms of population (e.g. hospitalized patients only vs primary care patients)
- Studies differ in terms of exposure definition (e.g. for example use different methods to ascertain common bacterial infections).
- Studies differ in terms of outcome measures (e.g. for example restricted to certain time-frames, or have a different definition of dementia or cognitive impairment).

4. Imprecision:

Low power and wide confidence intervals

- Serious imprecision: Wide confidence intervals
- Very serious imprecision: Very wide confidence intervals

5. Upgrading

Upgrading reasons include

<u>Large effect:</u>

- None: most effect estimates <2
- Strong association: effect estimates >2 or <0.5 (based on direct evidence, with no plausible confounders)
- Very strong association: effect estimates >4 or <0.2 (based on direct evidence with no serious problems with risk of bias or precision, i.e. with (sufficiently narrow confidence intervals).

<u>Plausible confounding</u>

- Would dilute the demonstrated effect: e.g. If, for instance, only sicker patients are exposed, yet they still fare better, it is likely that the actual exposure effect is even larger than the data suggest (confounding by indication).

- Would suggest spurious effect: When confounding is expected to increase the effect but no effect was observed.

6. Quality
Very low, low, moderate or high

Supplementary Material 4: Changes to Protocol

- In our protocol registered with the International Prospective Register of Systematic Reviews (PROSPERO), we had not included sepsis as one of the bacterial infections that we would be including in the present review. However, as sepsis is a common infection that fits our exposure definition. As such, excluding sepsis from the systematic review may result in the omission of relevant studies.

Risk of Bias

Supplementary Table 1. Risk of bias judgement, and justification
See Excel file.

Grade Quality Assessment

We planned to carry out a GRADE assessment on each infection and outcome, however, we could not assess the study quality when only one study was available. This was due to difficulties in assessing inconsistency and indirectness with a single study. As a result, study quality was assessed only for the studies investigating the association of sepsis or pneumonia with incident dementia.

Supplementary Table 2. GRADE assessment of quality of evidence for dementia outcome

		Downgrade				Upgrade	
Exposure	Study design and no. of studies	Risk of bias	Inconsistency	Indirectness	Imprecision		Quality
**Sepsis**	5 cohort studies and 1 case control study	serious	very serious	serious	serious	None	⊕◯◯◯ very low
**Pneumonia**	3 cohort studies	serious	very serious	serious	very serious	None	⊕◯◯◯ very low

Exploration of Heterogeneity
We explored heterogeneity on the association of sepsis with incident dementia by excluding studies from Taiwan.

Supplementary Figure 1. The effect of sepsis on dementia, by country (United States)

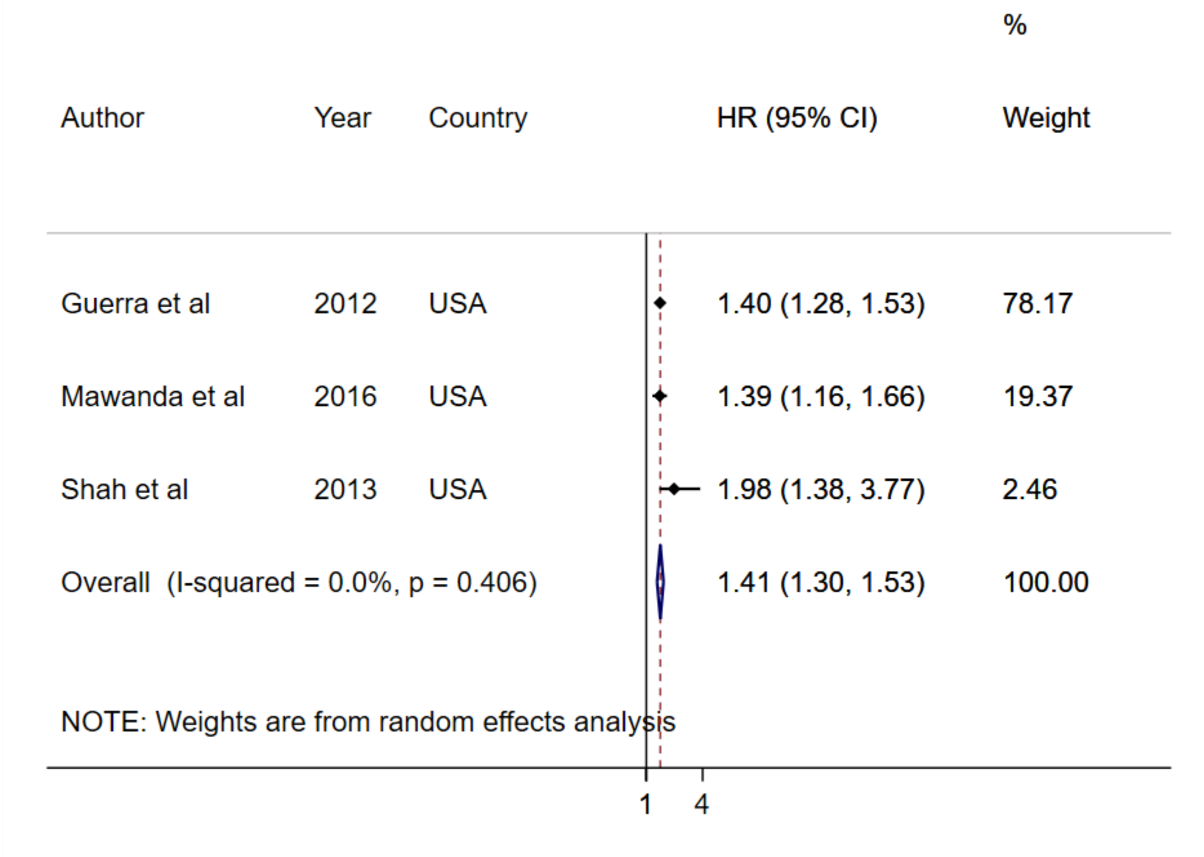

Supplementary Figure 2. The effect of sepsis on dementia after removing studies with a domain at high risk of bias

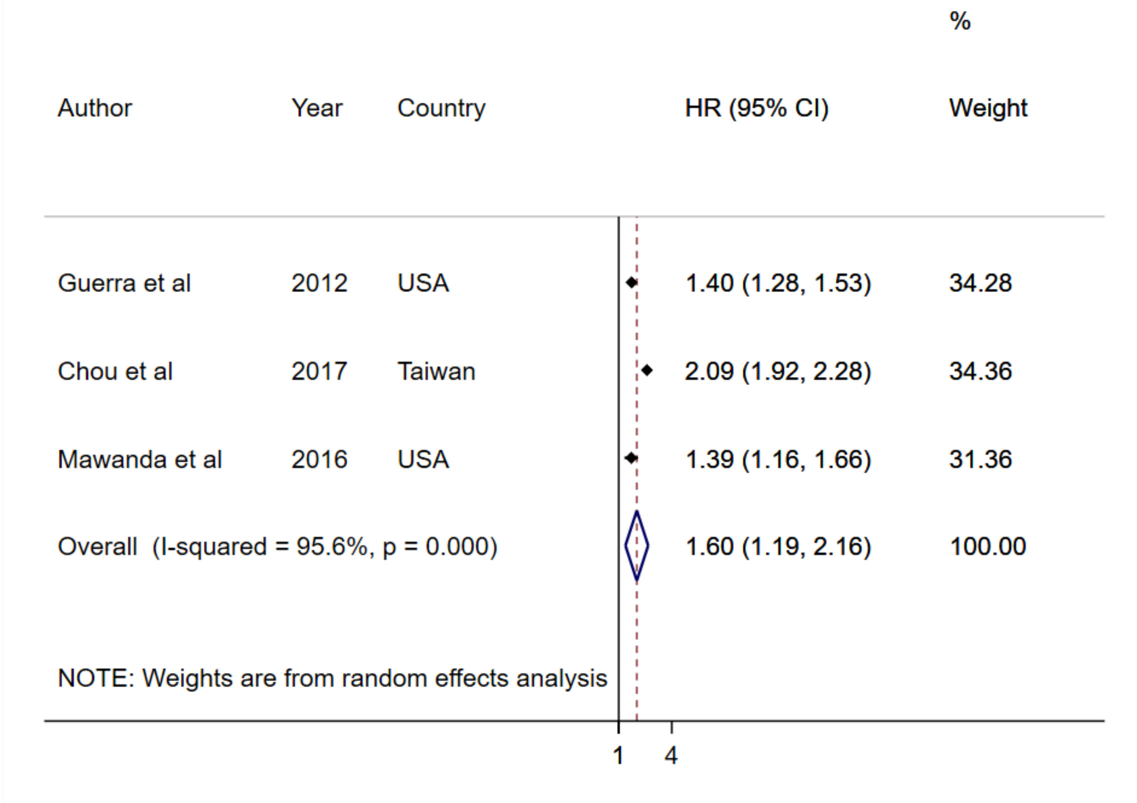

Sub Group Analyses

Supplementary Figure 3. The effect of sepsis on dementia, by age

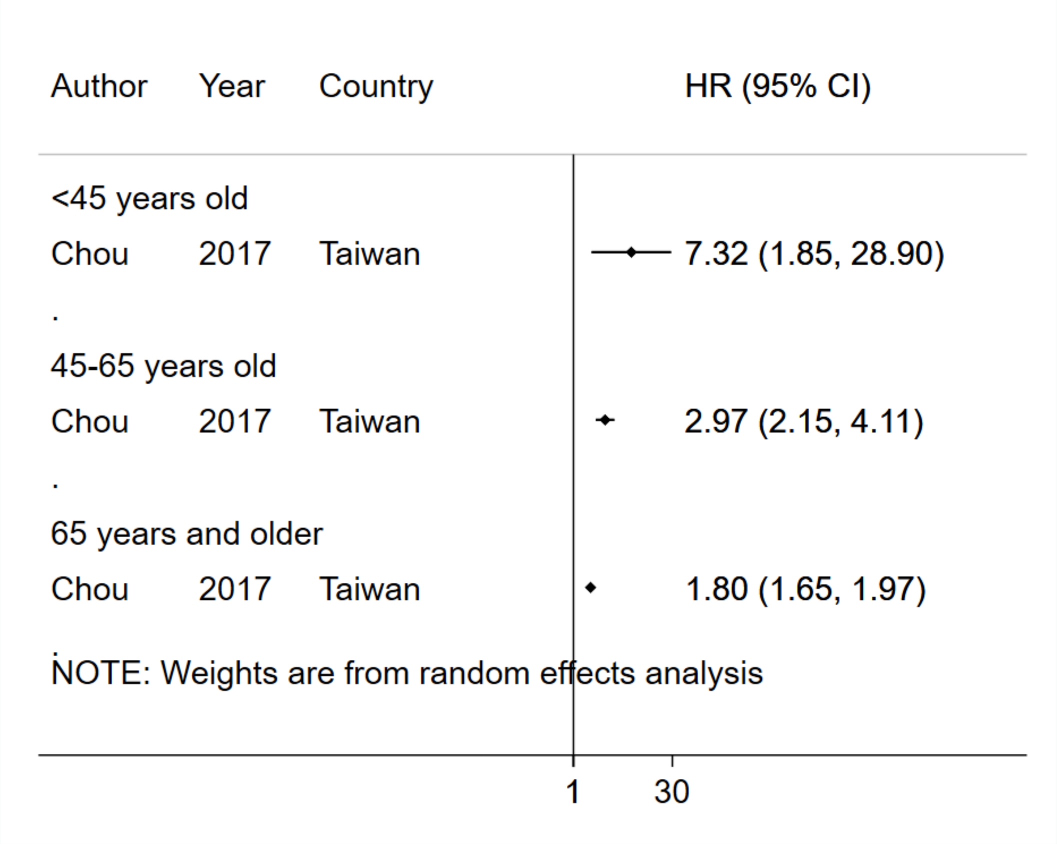

Supplementary Figure 4. The effect of sepsis on dementia, by sex

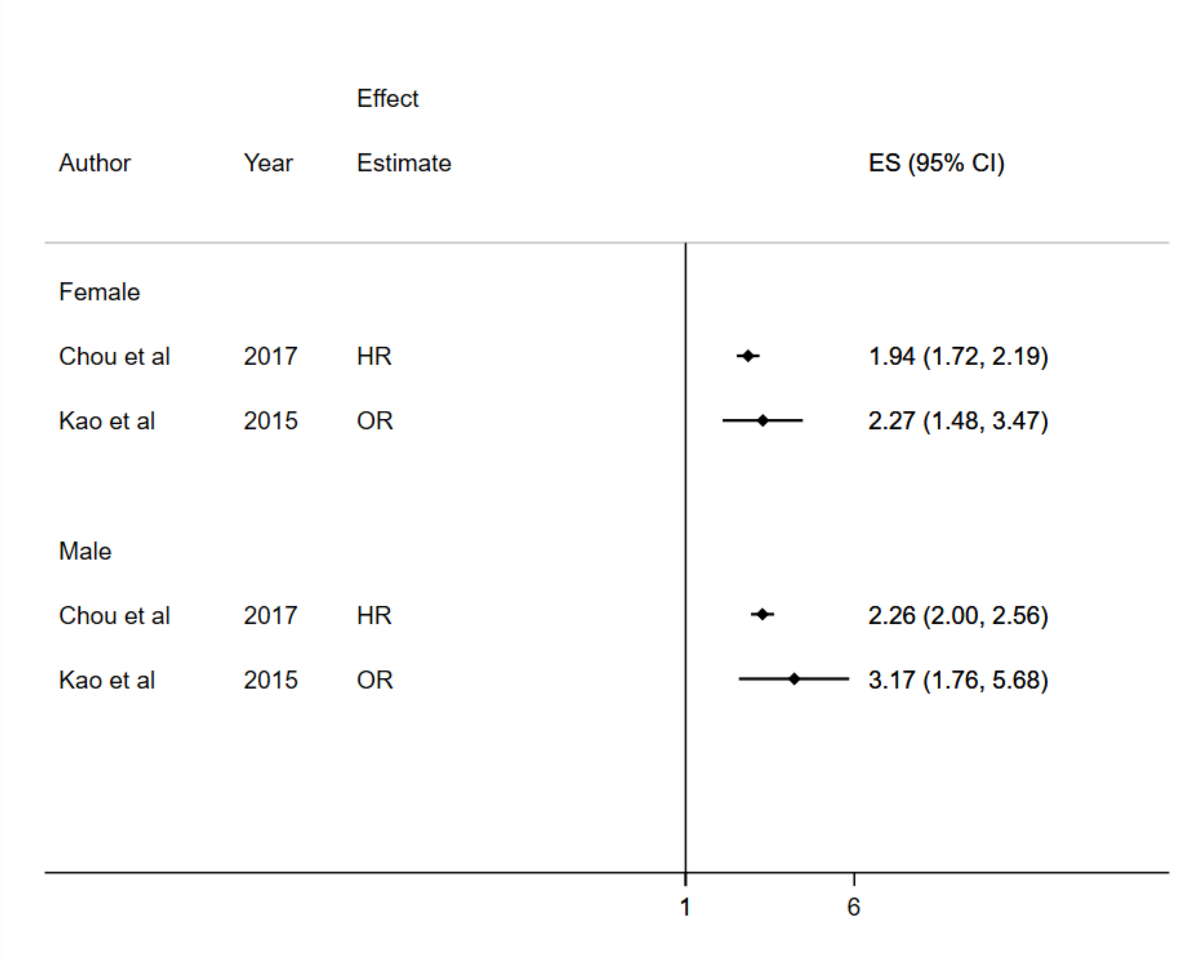

Supplementary Figure 5. The effect of sepsis on dementia, by dementia subtype

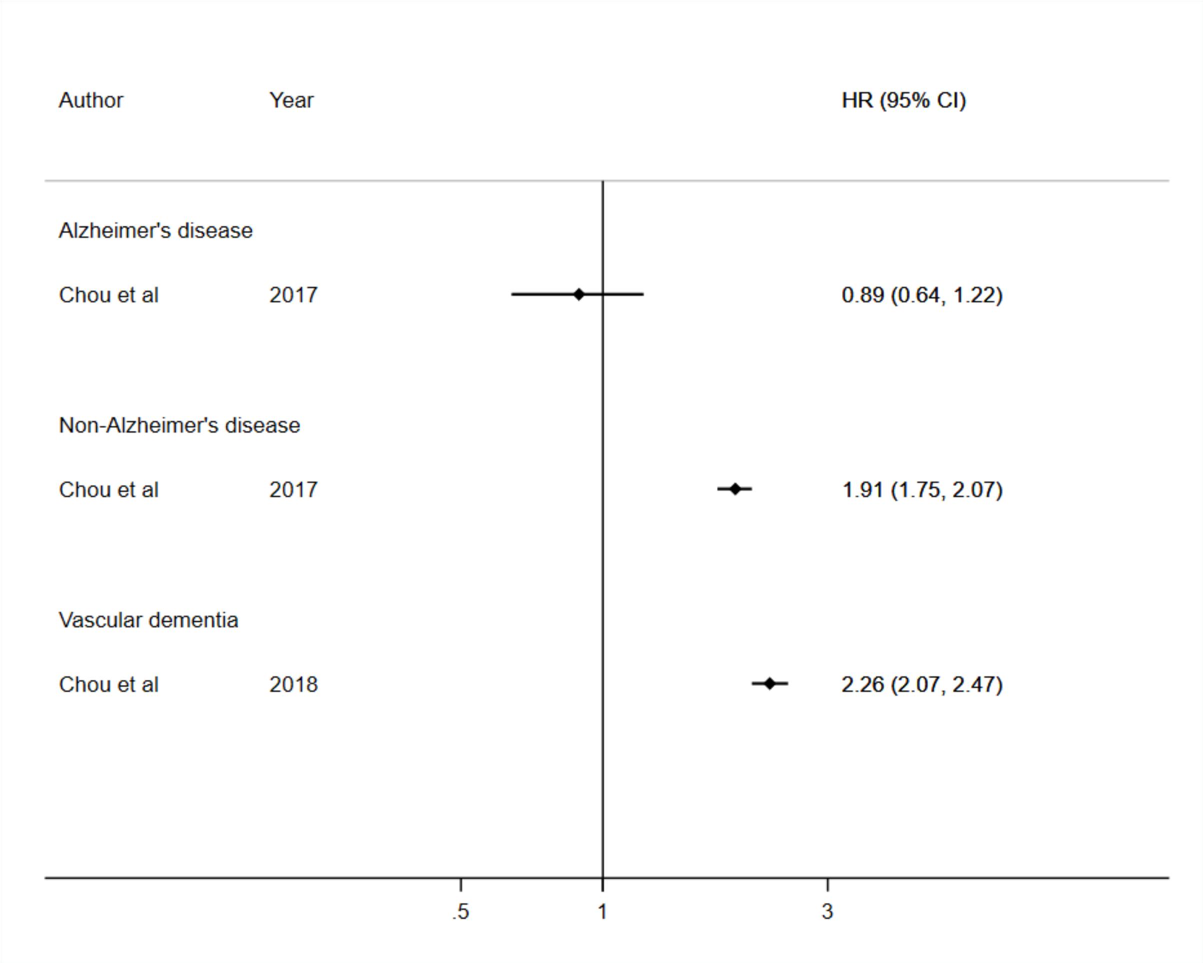

Supplement: Supplementary Material [file jad-76-jad200303-s001.pdf]
